# Investigating the Mechanical Properties and Durability of Asphalt Mixture Modified with Epoxidized Natural Rubber (ENR) under Short and Long-Term Aging Conditions

**DOI:** 10.3390/polym14214726

**Published:** 2022-11-04

**Authors:** Gailan Ismat Safaeldeen, Ramez A. Al-Mansob, Abdulnaser M. Al-Sabaeei, Nur Izzi Md Yusoff, Amiruddin Ismail, Wah Yen Tey, Wan Nur Aifa Wan Azahar, Ahmad Nazrul Hakimi Ibrahim, Taha Mohammed Jassam

**Affiliations:** 1 Kirkuk Technical Institute, Northern Technical University, Kirkuk 99W3+XMQ, Iraq; 2 Department of Civil Engineering, International Islamic University Malaysia, Jalan Gombak, Kuala Lumpur 50728, Malaysia; 3 Department of Civil Engineering, Faculty of Engineering, Thamar University, Dhamar 87246, Yemen; 4 Department of Civil Engineering, Faculty of Engineering and Built Environment, Universiti Kebangsaan Malaysia, Bandar Baru Bangi 43600, Malaysia; 5 Department of Mechanical Engineering, Faculty of Engineering, Technology, and Built Environment, UCSI University, Cheras, Kuala Lumpur 56000, Malaysia; 6 Malaysia-Japan International Institute of Technology, Universiti Teknologi Malaysia, Kampung Datuk Keramat, Kuala Lumpur 54100, Malaysia; 7 Department of Civil Engineering, UCSI University, Cheras, Kuala Lumpur 56000, Malaysia

**Keywords:** epoxidized natural rubber, natural polymers, aging, moisture damage, stiffness, durability, multivariable power least square method, asphalt

## Abstract

Modifiers such as fibers, fillers, natural and synthetic polymer extenders, oxidants and anti-oxidants, and anti-stripping agents are added to produce modified asphalt. However, polymers are the most widely utilized modifiers to enhance the function of asphalt mixtures. The objective of this research was to evaluate the mechanical properties and durability of epoxidized natural rubber (ENR)-modified asphalt mix under short- and long-term aging conditions. The physical and rheological characteristics of the base asphalt and ENR-modified asphalt (ENRMA) were tested. In order to evaluate the mechanical properties and durability of the modified mixtures, the resilient modulus of the ENR–asphalt mixtures under unaged, and short- and long-term aging conditions at various temperatures and frequencies was obtained. Furthermore, the resistance to moisture damage of asphalt mixtures was investigated. The findings showed that the stiffness of the ENR–asphalt mixes increased because of the mutual influence of short- and long-term aging on the mixes. In addition, ENR reduced the susceptibility to moisture damage. The stiffness of the mixes was influenced by the temperature and frequencies. By using mathematical modelling via the multivariable power least squares method, it was found that temperature was the dominant factor among all other factors. The results suggested that the durability of asphalt pavements is improved by using ENR.

## 1. Introduction

Researchers and the pavement industry usually modify base asphalt with polymers to improve their resistance to heavy traffic loadings and severe environmental conditions. Using one or more types of modifiers can alter the properties of asphalt, for instance, adhesive or cohesive strength and elasticity [1]. Among the agents frequently used are waxes, crumb rubber, polymers, acids and sulfur. Over time there is usually an increase in the demands made on roads and conventional asphalt reaches the limits of its performance, generally because of many factors such as an increase in temperature, rainfall variation, increase in tire pressure, axle weight and freight movement, which lead to higher maintenance requirements, and consequently, to an increase in the cost for road owners [2,3]. Other factors are the tendency to use thinner layers for pavements, the ever-increasing demand for cost savings and financial efficiency by reducing the frequency of maintenance, minimizing disruption to traffic flow and increasing the service life of the roads [4]. According to Ponniah and Kennepohl [5], polymer-modified asphalt (PMA) is cost-effective and does not exceed more than 100 percent of the cost of the base asphalt. Therefore, PMA can reduce the life cycle costs.

The ideal asphalt modifier will contribute to its low stiffness in cold surrounding temperatures, which can reduce cracking, while high stiffness at higher temperatures can reduce shoving and rutting and enhance the adhesion of the asphalt to the aggregate if there is moisture, to decrease the level of stripping [4,6]. The viscosity of modified asphalt changes dramatically at 60 °C, and the increase in viscosity will improve the binder–aggregate adhesion and also improve the resistance to plastic deformation. PMA can be applied wherever extra durability and performance are required because it improves the resistance to aging, thermal cracking, rutting, fatigue damage, stripping and temperature susceptibility. PMA can be used in all paving and maintenance applications, i.e., hot mix, cold mix, recycling, hot and cold crack filling, patching, chip seals and slurry seal [6,7].

An “elastomer” is a type of polymer with rubbery or elastic features that can return to its original size and shape if stretched and released. It can be regarded as elastic when the deformation is small (i.e., linear). Hence, an elastomer enables the binders to be more resilient and flexible [4]. Nevertheless, their influence on asphalt viscosity at various temperatures is accepted as their crosslinking occurs because of physical forces and can be reversed by increasing the temperature. Generally, elastomeric polymers have a greater influence on the properties of asphalt and therefore they are more suitable to be used with asphalts than plastomers. Examples of elastomeric polymers include ethylene-propylene-diene terpolymer, styrene isoprene styrene, styrene butadiene rubber, styrene-ethylene-butadiene, styrene-butadiene-styrene (SBS), natural rubber, epoxidized natural rubber and crumb tire rubber, which have been tested as asphalt modifiers [4,6,8]. Table 1 lists the advantages and disadvantages of using several popular polymers as modifiers. Natural rubber (NR) is a biopolymer that is extracted from trees, which has been used effectively over many decades in building and road construction as well as in the manufacturing of gloves and tires [9]. The use of NR as a bitumen modifier can promote sustainable pavement construction. NR has uncrosslinked long chain polymers that can be easily swollen, dispersed and absorbed by bitumen [10]. Overall, NR strengthens the stiffness and viscoelastic properties of bitumen; thus, it can resist more strain at high temperatures [11]. On the other hand, NR-modified bitumen is known for its poor adhesion properties and poor weather resistance, which have a direct effect on the long-term performance of rubberized asphalt pavement [12]. Yousefi [13] studied the natural rubber-modified asphalt binders and discovered that they have better intermediate and high performance when compared to base asphalt. The enhanced characteristics include a lower degree of penetration and a higher point of softening. On the other hand, there is an improvement in the low temperature properties, for example, the Frass breaking point. Asphalt can easily absorb NR and cause swelling. However, a decrease in workability is one of issues that can be resolved by mixing NR with asphalt, which can be done by adding warm mix additives. According to previous studies, the optimum content of NR for asphalt is 4 to 6% by weight of asphalt [10]. It has been demonstrated that the properties of binders could be improved significantly and economically by using 3 to 6% polymer as a modifier [14]. The most important goal in selecting a modifier is to make the asphalt less stiff in cold weather, and stiffer or more viscous in hot weather. High-performing modified asphalt should also have improved fatigue resistance and water sensitivity.

The mechanical properties and durability of NR-modified asphalt mixtures have been investigated in the literature [15,16,17]. It was reported that the addition of NR enhanced the rutting performance of asphalt mixture compared to unmodified mixtures and the 6% NR resulted in promising improvements [16,17]. In addition, Prastanto et al. [18] found that the 5–7% NR improved the mechanical properties of asphalt mixtures. In contrast to the aforementioned advantages of NR, it was reported that NR-modified bitumen, known for its poor adhesion properties and poor weather resistance, led to a deterioration in the durability of asphalt mixtures due to the segregation between the NR-modified bitumen and the aggregate particles [11,12]. These phenomena are mostly dependent on the type of NR used and the presence of water, which has a direct effect on the long-term performance of rubberized asphalt pavement [12].

Epoxidized natural rubber (ENR) was first utilized in the rubber industry in the 1980s [11]. ENR is made by a chemical modification of natural rubber formed by reacting proxy formic acid and natural rubber [11]. This material has excellent mechanical characteristics, a high glass transition temperature, and high strength due to its capacity to carry strain crystallization. Higher oil resistance, damping, high adhesion, and decreased gas permeability are all accelerated by these characteristics [35,36]. ENR is said to increase the viscosity and rigidity of binders while lowering their temperature susceptibility. Furthermore, the optimal level of the modification in the binder was reported to be 6% ENR [12,34,37,38]. Therefore, the ENR was employed in this research to eliminate some of the aforementioned NR disadvantages at different environmental conditions, including short- and long-term aging.

The aging of the asphalt has a major impact on the roadway structure’s durability. The loss of volatile compounds and oxidation of the asphalt during the production of asphalt mix (short-term ageing) and gradual oxidation over the service life in the field (long-term ageing) are the two main causes of ageing [39,40]. When asphalt comes into contact with oxygen (air), it progressively reacts with oxygen, increasing the viscosity and making it harder and less flexible. The percentage of viscosity is greatly influenced by temperature, time, and the thickness of the asphalt coating. Extreme age hardening can result in brittle asphalt with considerably reduced flow capacities, limiting the asphaltic mixes’ capacity to sustain traffic and thermally-generated stresses and strains, which can lead to various types of cracking [4]. Therefore, this research aimed to investigate the performance of unaged and aged base asphalt mixtures (HMA-0) and an asphalt mixture modified with 6% ENR (HMA-6) by weight of asphalt, in terms of the indirect tensile resilient modulus at various temperatures and frequencies, and its moisture susceptibility.

## 2. Materials and Methods

### 2.1. Materials

The asphalt employed in this research has a penetration grade of 80/100 and was supplied by the asphalt factory in Port Klang, Malaysia with a specific gravity of 1.03. ENR 50, with 53 percent epoxidization, was purchased from the Malaysian Rubber Board, Malaysia and measured 2.36 mm (prior to shearing). Table 2 lists the physical properties of the materials.

The optimal level of the modification in the binder was found to be 6% ENR, therefore this research investigated the performance of a base asphalt mixture (HMA-0) and an asphalt mixture modified with 6% ENR, by weight of asphalt. This 6% ENR was also reported in our previously published work as an optimum [38]. The basic properties of the base and 6% ENR-modified asphalt binders were obtained and compared as shown in Table 3. These results fulfilled the Superpave specifications that are required for rutting and fatigue distress resistance [6,41].

### 2.2. Preparing the Modified Bitumen and Asphalt Mixes

The ENR-modified asphalts were made by mixing 6% of ENR (by weight of the base asphalt) with the base asphalt for 60 min in a high shear mixer at a temperature of 160 °C (±1 °C) at a speed of 4000 rpm [12]. After the temperature stabilized at 160 °C, ENR was added to the asphalt. 

**Figure 1:**
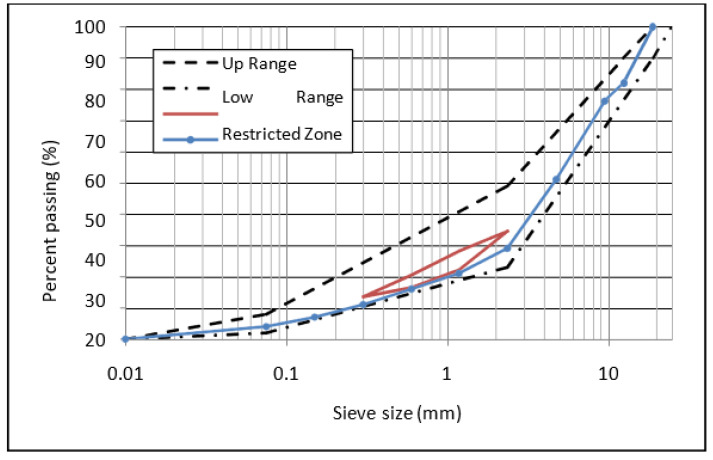
Aggregate gradation.

The asphalt mixes were developed according to the Superpave mix design standard [42]. Asphalt mixes were designed with equivalent single-axle loads (ESALs) less than 10^7^. For each modified and unmodified HMA, mixes with five percentages of asphalt content ranging from 4 to 6% with a 0.5% increment were produced (with three replications for every sample). For the modified and unmodified HMA, specimens were produced using blended mineral aggregates with 0.5 percent binder by weight of aggregate. The samples were compacted using a Superpave gyratory compactor (SGC) with 4% air voids, the optimal binder content for achieving acceptable volumetric characteristics as compared to the specified mix requirements. The nominal maximum particle size of aggregate was chosen to be 19 mm. Figure 1 shows the gradation of the mixture adopted in this study. The design data for the asphalt mix was taken from an earlier publication by Al-Mansob et al. [34].

### 2.3. Indirect Tensile Resilient Modulus Test

In this study, the testing procedures were conducted by utilizing the Universal Testing Machine in accordance with the ASTM-D4123 standard. The resilient modulus (M_R_) is a basic and important metric that researchers use to find the mechanical properties of asphalt mixes, which can be employed in the mechanical design of asphalt pavement structures. This is a non-destructive test that measures the ratio of used stress to recoverable strain at a different frequency and temperature.

The gyratory compactor was used to compact samples with a diameter of 100 mm and a height of 63.5 mm for every type of mix, to obtain a 4% air void. Prior to the testing, asphalt mixes and testing equipment were conditioned in the environmental chamber for at least two hours at the testing temperatures. After the testing was underway and the data was recorded, the sample was set with a haversine wave pattern with five counts of conditioning pulses followed by five loading pulses. In addition, the load was programmed to have a pulse width of 100 ms and a pulse repetition period of 3000 to 1000 ms. According to Tayfur et al. [43], a pulse repetition cycle of 1000 ms replicates a heavy traffic load on the pavement, while 3000 ms simulates a low traffic load. Table 4 lists the test parameters.

### 2.4. Durability of Asphalt Mixes

A durable product is one that can tolerate repeated use without considerable deterioration. Engineers limit the term durability when it comes to bituminous paving materials to those impacts that are related to the environment, such as moisture and ageing, providing that the bituminous pavement layer is built correctly and according to the standards. Durability was also defined as the ability of the materials in the mix to withstand the effects of water, ageing, and temperature fluctuations for an extended period of time in the context of particular traffic loads [44]. However, because the expense of maintaining and rehabilitating pavement structures is high, durability must be considered during the material selection, design, and implementation stages. Therefore, the two most major damage variables affecting durability, moisture damage and age hardening were considered in this study.

#### 2.4.1. Moisture Susceptibility

It is critical to determine the design mix’s moisture sensitivity. This test was conducted in accordance with AASHTO-T283 [45]. Samples of the unmodified and ENR-modified asphalt mixtures, with a diameter of 100 mm and a height of 63.5 mm, were compacted until they had an air void of 7%. The samples were divided into a control and a conditioned subset. At 60 °C, the conditioned subset was exposed to partial vacuum saturation, a freeze cycle, and a 24 h thaw cycle. The indirect tensile strengths of all samples were tested. The moisture sensitivity was calculated as a ratio of the conditioned subset’s tensile strengths divided by the dry (control) subset’s tensile strengths. A minimum of 80% maintained tensile strength is required. 

#### 2.4.2. Age Hardening

In order to imitate hardening throughout the mixing, the short-term ageing of the mixes usually entails heating the loose mix in an oven before compaction. The AASHTO-R30 standard was used to perform short-term ageing. The technique calls for a loose mix to be aged in a forced draught oven for four hours before compaction at a temperature of 130 °C or a temperature proportional to the required compaction temperature, whichever is greater, with stirring once every hour. To analyze the changes in performance due to ageing, an indirect tensile modulus test was performed on samples before and after the short-term oven ageing.

Long-term ageing was conducted in accordance with the procedures of the SHRP-A-383 [46]. To assess the performance changes caused by long-term oven ageing, the indirect tensile modulus test was performed on samples before and after the long-term oven ageing. Depending on the intended service life of the bituminous mix, several time periods were adopted for the long-term oven ageing. A two-day oven ageing time appears to replicate up to five years of service, whereas a five-day ageing period is utilized to replicate the ageing process in ten-year projects.

A long-term ageing test was performed on the compacted mixtures that had already completed the short-term oven ageing process. Samples were compacted and baked for 120 h at 85 °C in a forced draught oven (5 days). The oven was turned off at the completion of the ageing time and allowed to cool to room temperature before the samples were removed. The samples were not tested for at least 24 h after they had been taken out of the oven. The testing procedure that was used for the tests was the same as for the unaged conditioned samples.

### 2.5. Mathematical Modelling

The multivariable power least square method (MPLSM) that was recently formulated by Tey et al. [47] was used to model the experimental data. The normalized MPLSM was applied by converting the factors of frequency ***f*** and temperature ***T*** into normalized frequency ***f*** and normalized temperature ***T*** respectively:

(1) $$f=C+\frac{f-f_{\min}}{f_{\max}-f_{\min}},$$

(2) $$T=C+\frac{T-T_{\min}}{T_{\max}-T_{\min}},$$

where ***C*** is an integer. In our case, we applied ***C*** = 0.5. In MPLSM, the factors were correlated with the output via the power function, as shown in Equation (3).

(3) $$y^{h}=af^{b_{1}}T^{b_{2}},$$

Upon simplification, the indexes *b*_1_ and *b*_2_ can be obtained by solving the following matrix:

(4) $$\left(\begin{array}{cc} \gamma_{1} & -\lambda_{12}\\ -\lambda_{21} & \gamma_{2} \end{array}\right)\left(\begin{array}{c} b_{1}\\ b_{2} \end{array}\right)=\left(\begin{array}{c} \xi_{1}\\ \xi_{2} \end{array}\right),$$

(5) $$\gamma_{j}=n\left\{\sum_{i=1}^{n}\sum_{j=1}^{2}{\left[\mathrm{In}\left(x_{j,i}\right)\right]}^{2}\right\}-{\left\{\sum_{i=1}^{n}\sum_{j=1}^{2}\left[\mathrm{In}\left(x_{j,i}\right)\right]\right\}}^{2},$$

(6) $$\xi_{j}=n\left[\sum_{i=1}^{n}\sum_{j=1}^{2}\mathrm{In}\left(x_{j,i}\right)\mathrm{In}\left(y_{j}\right)\right]-\left[\sum_{i=1}^{n}\sum_{j=1}^{2}\mathrm{In}\left(x_{j,i}\right)\right]\left[\sum_{i=1}^{n}\mathrm{In}\left(y_{j}\right)\right],$$

(7) $$\lambda_{j,k}=\left[\sum_{i=1}^{n}\sum_{j=1}^{2}\mathrm{In}\left(x_{j,i}\right)\right]\left[\sum_{i=1}^{n}\sum_{j=1}^{2}\mathrm{In}\left(x_{k,i}\right)\right]-n\left[\sum_{i=1}^{n}\sum_{j=1}^{2}\mathrm{In}\left(x_{j,i}\right)\mathrm{In}\left(x_{k,i}\right)\right],$$

where *x* = [ ***f*** ***T*** ] and *n* is the number of available data in each factor. Meanwhile the constant *a* can be found via Equation (8).

(8) $$a=\exp\left\{\frac{1}{n}\left(\left(\sum_{i=1}^{n}\mathrm{In}\left(y_{i}\right)\right)-\sum_{j=1}^{2}\left(b_{j}\sum_{i=1}^{n}\sum_{j=1}^{2}\mathrm{In}\left(x_{j,i}\right)\right)\right)\right\},$$

**Figure 2:**
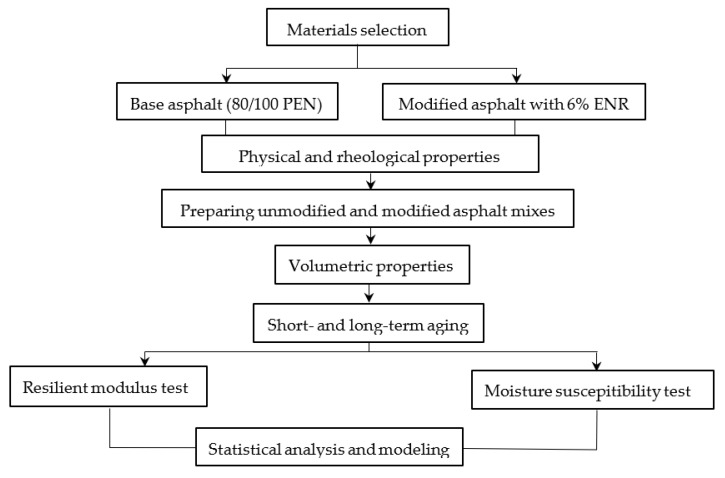
Flow chart of the research.

A flow chart of the experimental design and data analysis is presented in Figure 2.

## 3. Results and Discussion

The effect of the 6% ENR content on the mechanical performance of the ENR-modified asphalt mix (HMA-6) was reported and compared with the samples of the base asphalt mixes (HMA-0). In addition, the effects of the ageing on HMA-6 and HMA-0 were investigated. The HMA design was based on the Superpave volumetric mix design. The difficulty in HMA performance testing, as with asphalt binder characterization, is to create physical tests that can properly characterize important HMA performance characteristics and how these parameters evolve throughout the lifetime of a pavement. These key parameters include stiffness and moisture susceptibility [44].

### 3.1. Volumetric Properties of Mixes

**Figure 3:**
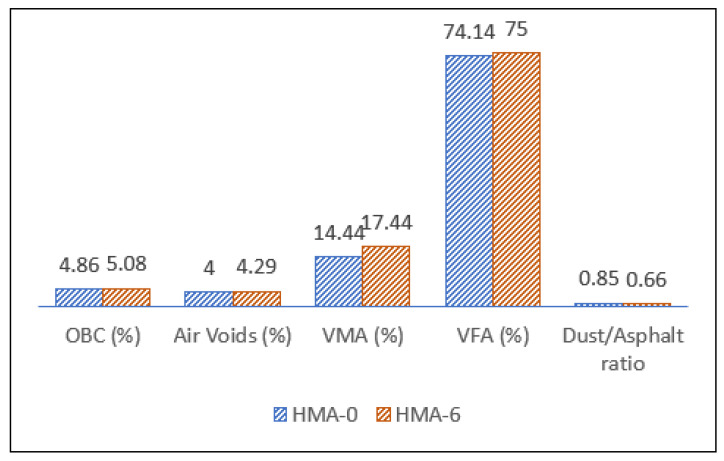
Volumetric properties of the mixes.

The optimal binder content (OBC), voids in mineral aggregate (VMA) effective binder content (Pbe), voids filled with asphalt (VFA), and the dust to effective binder content ratio (P0.075/Pbe) are among the volumetric characteristics. The VMA, VFA, air voids, and dust percentage were the most important factors in determining the characteristics of Superpave HMA mix. The volumetric characteristics of the design mixes related to the OBC of the mix, as well as the mix design criteria are shown in Figure 3. The findings revealed that both of the mix characteristics met all of the Superpave system’s requirements.

### 3.2. Resilient Modulus Test Results

The resilient modulus is a key parameter for assessing pavement responsiveness in terms of dynamic loads and strains. The modulus of asphalt is one of the main design parameters required when using elastic-layered system theory to build asphalt pavements [48,49]. Horizontal deformations were determined on both sides of the sample during testing, and the resilient modulus was computed as a result. This test was used to compare and characterize all of the samples (unmodified, modified, unaged and aged HMAs), which were evaluated at temperatures of 5, 25, and 40 °C and at frequencies of 0.33, 0.5, and 1 Hz. Regardless of the presence of the modifier, the resilient modulus of all mixtures increased when the temperature decreased. The decrease in the resilient modulus was noted in previous studies that used various polymers as modifiers [43].

**Figure 4:**
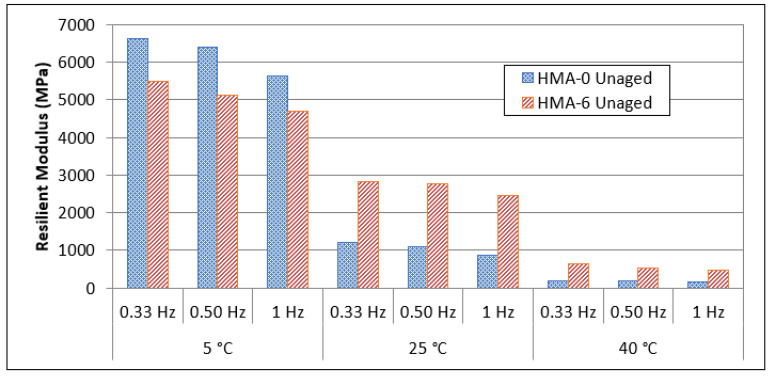
Resilient modulus of unaged HMA mixes.

For the unaged mixes and at low temperature (5 °C), the resilient modulus values were higher for the HMA-0 mixes compared to the HMA-6 mixes. This indicates that the elasticity decreased at low temperature when the polymer was used as a modifier, as shown in Figure 4. On the other hand, the results show that as the pulse repetition frequency through the loading time change from 0.333 to 1 Hz, the resilient modulus values decrease. Nevertheless, HMA-0 shows the highest resilient modulus at low frequency loading and a temperature of 5 °C.

A general trend was found in that the ENR modifier had a similar effect on the resilient modulus values of HMA-6 mixes at 25 and 40 °C for all loading frequencies, where the resilient modulus for HMA-6 increased compared with that of the HMA-0. However, the resilient modulus decreased at all temperatures and with an increase in the loading frequency. The resilient modulus can be used as a tool to evaluate the performance of asphalt pavement for low temperature cracking, fatigue, and permanent deformation [50,51,52]. Finally, these results indicate that the HMA-0 is more susceptible to low temperature cracking because of the increase in the resilient modulus at low temperatures, it is more susceptible to fatigue cracking because of the decrease in the resilient modulus at intermediate temperatures, and more susceptible to permanent deformation because of the decrease in the resilient modulus at high temperatures.

Various types of polymers have been used as modifiers and it was reported that the control asphalt mix has a higher resilient modulus than the modified asphalt mixes at low temperature (5 °C), which means a higher elasticity modulus (stiffness), and thus, a lower cracking resistance [43]. On the other hand, the resilient modulus of modified asphalt mixes is increased compared to unmodified mixes at intermediate and high temperatures (25 and 40 °C). Overall, the results indicate that the resilient modulus of the base mixes, especially at low temperature (5 °C), are greater than the polymer-modified asphalt mixes. However, at intermediate and high temperatures (25, 40 °C) the values tend to decrease.

**Figure 5:**
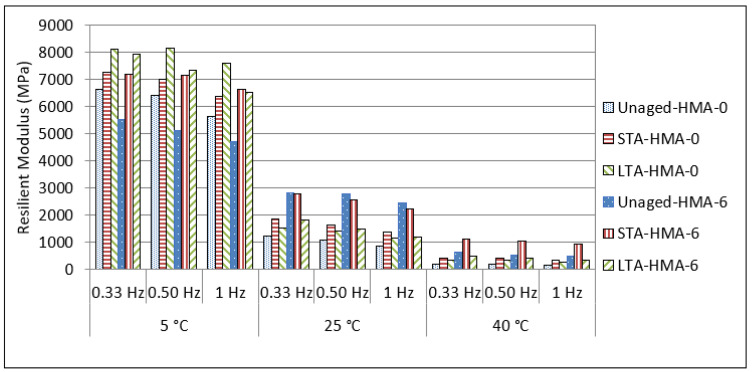
Resilient modulus of unaged and aged HMA mixes.

**Figure 6:**
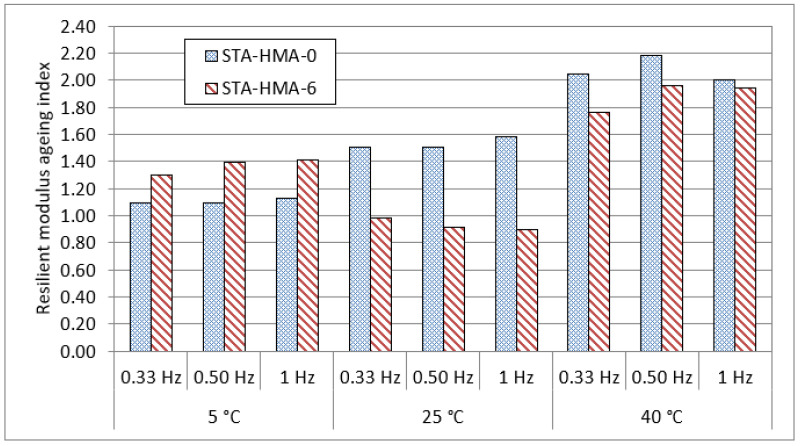
Resilient modulus ageing index of short-term aged mixes.

**Figure 7:**
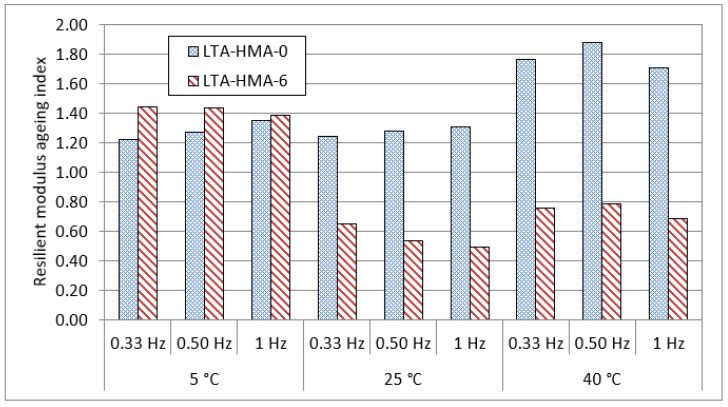
Resilient modulus ageing index of long-term aged mixes.

Figure 5 shows the resilient modulus test results for the HMA-0 and HMA-6 mixes that were subjected to ageing. For short-term ageing (STA), mixes were placed in an oven at 135 °C for HMA-0 and at 150 °C for HMA-6 for 4 h. These temperatures are known as compaction temperatures, and were taken from the viscosity results. On the other hand, for long-term ageing (LTA), mixes were placed in an oven at 85 °C for 5 days. Overall, it can be observed that the resilient modulus of the ENR-modified asphalt mixtures, especially at the intermediate and higher test temperatures, is higher compared to unmodified asphalt mixtures over the different frequencies adopted in this study. These results could be attributed to the higher stiffness and elasticity of ENR-modified asphalt binders compared to the unmodified asphalt binders. It can be also seen that after STA and LTA, ENR as a modifier had a significant, positive effect on the resilient modulus values of the HMA mixes at all temperatures, particularly for the LTA mixes. This could be attributed to the resistance of ENR to aging and its lower susceptibility to temperature. In order to explore the reasons behind the reduction in the resilient modulus after the aging, the resilient modulus ageing index of the STA and LTA mixes were calculated as shown in Figure 6 and Figure 7, where the ageing index for every mix was calculated based on the following equation:

(9) $$\mathrm{Ageing}\;\mathrm{index}=\frac{M_{R\left(\mathrm{unaged}\;\mathrm{mix}\right)}}{M_{R\left(\mathrm{aged}\;\mathrm{mix}\right)}},$$

where $M_{R\left(\mathrm{unaged}\;\mathrm{mix}\right)}$ is the resilient modulus ageing index for the unaged mixes and $M_{R\left(\mathrm{aged}\;\mathrm{mix}\right)}$ is the resilient modulus ageing index for the STA or LTA mixes.

At 5 °C, as shown in Figure 6 and Figure 7, the resilient modulus ageing values of HMA-6 after ageing showed similar trends to HMA-0, indicating that the HMA-6 mixes are less susceptible to cracking at low temperatures than HMA-0 for the short- and long-term aging period. On the other hand, at LTA, the resilient modulus ageing index for HMA-6 decreased with the increase in the frequency loading, while it increased for the HMA-0. At 25 °C, the resilient modulus ageing values of HMA-6 after ageing showed similar trends to HMA-0, where the resilient modulus ageing values for the HMA-6 mixes significantly decreased compared with HMA-0, indicating that the HMA-6 mixes are less susceptible to fatigue cracking for the short- and long-term period. At 40 °C, the resilient modulus ageing values of HMA-6 after ageing showed similar trends to HMA-0, where the resilient modulus ageing values for the HMA-6 mixes significantly decreased compared to HMA, indicating that the HMA-6 mixes are less susceptible to rutting for the short- and long-term period. These findings also reveal that the ENR-modified asphalt mixtures are highly resistant to aging due to the addition of ENR compared to the unmodified asphalt mixtures.

### 3.3. Moisture Suscepitibility Results

**Figure 8:**
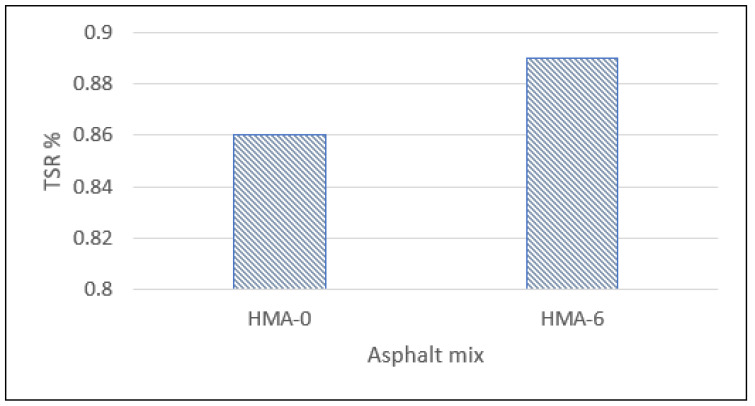
Moisture susceptibility test results.

The indirect tensile strength (ITS) of each mix for the dry and conditioned cases was found and these were averaged based on the results of three replicates according to the AASHTO-T283 standard. Figure 8 shows the results of the ITS test. All samples were prepared to contain air voids within the range of 7 ± 0.5%, according to the AASHTO standard. The trend indicated that the ITS of all specimens decreased from the dry to conditioned specimens, indicating that the mixes had deteriorated, which affected the HMA mix’s strength.

The tensile strength ratio (TSR) indicates the HMA mix’s sensitivity to moisture damage. The HMA-0 and HMA-6 mixes fulfilled the necessary minimum TSR value of 80 percent as required by the AASHTO standard. The indirect tensile strength tests indicated that the HMA-6 mixtures had greater tensile strength compared with the unconditioned and conditioned HMA-0, indicating that HMA-0 is more sensitive to moisture. This finding agrees with the literature in that styrene-butadiene-styrene (SBS) decreased HMA’s sensitivity to moisture damage [53]. In addition, in the same study, it was found that modified-asphalt mix samples achieved a greater TSR value than the base HMA, by 89% and 77%, respectively.

### 3.4. Modelling Results

Based on the results in Table 5, it can be seen that all the unaged HMA-0, LTA-HMA-0, and STA-HMA-0 were more sensitive to the temperature and less sensitive to frequency. Similarly, it can be seen that by comparing the results that the unaged HMA-6, STA-HMA-6, and LTA-HMA-6 were more sensitive to the temperature and less sensitive to frequency. In general, it is obvious that temperature is the dominant factor in all cases. The proposed equations shown in Table 5 can be successfully used for predicting the effects of aging on the base and modified asphalt mixtures with an adequate degree of accuracy considering the limits applied in this research.

The index ratio = *b*_2_/*b*_1_, which represents the sensitivity weightage of temperature against frequency.

## 4. Conclusions

This study evaluates the performance of unaged, short- and long-term aged, unmodified and modified asphalt mixes with ENR of 6%, in terms of the indirect tensile resilient modulus at temperatures of 5, 25 and 40 °C and load frequencies of 0.33, 0.5 and 1 Hz, and their susceptibility to moisture. Based on the findings, the following conclusions are highlighted.

1. The characteristics of the ENR-modified asphalt mixes in terms of the bearing capacity showed that adding ENR results in a decrease in the resilient modulus at low temperature, and an increase in the resilient modulus at intermediate and high temperatures.
2. Overall, in term of the durability of the ENR-modified asphalt mixes, the influence of ageing on the resilient modulus was found to have a similar effect at low, intermediate and high temperatures for the base and the modified HMA.
3. The resilient modulus after STA was higher or close to the resilient modulus of unaged mixtures at low, intermediate, and high temperatures; however, there was a reduction after the LTA at intermediate and high temperatures, which was clearly observed in ENR-modified asphalt mixtures, indicating the resistance of ENR-modified asphalt mixtures to LTA compared to unmodified mixtures.
4. Based on the effect of the ageing index on the resilient modulus results, it is clear that ENR reduced the ageing effect at all intermediate and high temperatures and frequencies considered in this study, indicating that the modified mixtures were less susceptible to temperature compared to the unmodified mixtures.
5. ENR-modified asphalt mixes showed higher tensile strength compared to control, indicating that ENR-HMA is less sensitive to moisture damage than base-HMA. Although the improvement was minor, the findings are still within acceptable limits based on the AASHTO standards.

## Figures and Tables

**Table 1:** Popular polymers for modification of asphalt.

Polymer	Advantages	Disadvantages	References
SBS	Increase stiffness—reduce temperature susceptibility—improve rutting resistance—improve fatigue resistance—moisture susceptibility.	Expensive—workability	[19,20,21,22,23,24]
Ethylene-vinyl acetate (EVA)	Increase stiffness—reduce temperature susceptibility—improve rutting resistance—improve fatigue resistance—good storage stability.	Workability—low elastic recovery	[22,25,26]
Styrene butadiene rubber (SBR)	Increase stiffness—reduce temperature susceptibility—improve rutting resistance—improve fatigue resistance—moisture susceptibility.	Low elastic recovery—workability	[22,27,28]
NR	Increase stiffness—low cost—improve the temperature susceptibility.	Low elastic recovery—poor compatibility with asphalt—workability—low temperature properties	[10,13,27,28]
Polyethene (PE)	Low cost—Good high temperature properties—increase stiffness—moisture susceptibility.	Limited improvement in elasticity—poor compatibility with asphalt	[22,29,30,31,32,33]
Polypropylene (PP)	Low cost—increase stiffness—moisture susceptibility.	Limited improvement in elasticity—poor compatibility with asphalt	[22,32,33]
Ethylene-butyl acrylate (EBA)	Increase the rutting resistance	Limited improvement in low temperature properties	[22]
Styrene-isoprene-styrene (SIS)	Temperature sensitivity is reduced, and elastic responsiveness is improved.	Low resistance to oxidation—high cost	[22]
ENR	Low cost—improve the temperature susceptibility—increase in the resistance to rutting.	Poor compatibility with asphalt	[12,34]

**Table 2:** Properties of the materials.

Material	Properties	Value
Asphalt	Specific Gravity	1.03
	Softening point, °C	45.7
	Penetration, 0.1 mm	82 @ 25 °C
	Viscosity, Pa.s	12.60 @ 80 °C
	Ductility, cm @ 5 cm/min	>100 @ 25 °C
	Ductility, cm @ 5 cm/min	20 @ 10 °C
ENR	Specific gravity	0.9366
	Size, mm (prior to shearing)	2.36
	Modulus at 100%, MPa	0.74
	Modulus at 300%, MPa	1.56
	Tensile strength, MPa	28.3
	Elongation at break, %	770
	Hardness, IRHD	36
	Tension fatigue, ring 0–100% extn, kcs	234
	Tension fatigue, ring 50–150% extn, kcs	880
	Tear strength, trouser, N/mm	6.3

**Table 3:** Binder properties.

	Parameter	Base Asphalt	ENRMA6
Original binder	Softening point, °C	45.7	53.8
	Penetration, 0.1 mm	82	64
	Viscosity @ 135 °C, Pa.s	0.24	0.61
	Flash point, °C	275	329
	Dynamic Shear at 10 rad/s (G*/sinδ), kPa	0.18	2.10
Rolling thin film oven test (RTFOT)	RTFOT Weight Loss, %	0.36	0.29
	Dynamic Shear at 10 rad/s (G*/sinδ), kPa	0.97	3.83
Pressure aging vessel (PAV)	Dynamic Shear at 10 rad/s (G*/sinδ), kPa	3962	2414

**Table 4:** Indirect tensile resilient modulus parameters.

Parameter	State
Temperatures	5, 25 and 40 °C
Loading pulse width	100 ms
Pulse repetition periods	1000, 2000 and 3000 ms
Applied load	Produced 10% of the indirect tensile strength

**Table 5:** Modelling results.

Case	Modelled Equation	R^2^	RMSE	Index Ratio
Unaged HMA-0	$y^{h}=812.3960\times f^{-0.2033}T^{-3.0653}$	0.9888	598.8	15.077
STA-HMA-0	$y^{h}=1295.8519\times f^{-0.1892}T^{-2.5098}$	0.9872	579.5	13.265
LTA-HMA-0	$y^{h}=1192.5361\times f^{-0.1740}T^{-2.8079}$	0.9871	585.9	16.137
Unaged HMA-6	$y^{h}=1649.9469\times f^{-0.1731}T^{-1.8314}$	0.8618	927.8	10.580
STA-HMA-6	$y^{h}=2604.0652\times f^{-0.1477}T^{-1.666}$	0.9818	424.0	11.280
LTA-HMA-6	$y^{h}=1279.9825\times f^{-0.2991}T^{-2.5275}$	0.9888	518.6	8.450

## Data Availability

The data presented in this study are available on request from the corresponding author.
